# Impaired episodic memory in PTSD patients — A meta-analysis of 47 studies

**DOI:** 10.3389/fpsyt.2022.909442

**Published:** 2022-09-28

**Authors:** Maria Petzold, Nico Bunzeck

**Affiliations:** ^1^Department of Psychology, University of Lübeck, Lübeck, Germany; ^2^Center of Brain, Behavior, and Metabolism, University of Lübeck, Lübeck, Germany

**Keywords:** episodic memory, PTSD, meta-analysis, trauma, stress

## Abstract

Episodic memory impairments beyond the traumatic event might be a characteristic hallmark of post-traumatic stress disorder (PTSD). Although several studies support such a claim, empirical findings are inconsistent. Therefore, we performed a random-effects meta-analysis including data from a total of 47 studies and 3,062 subjects. As main finding, we can show that PTSD patients show episodic memory deficits compared to all controls. This effect was significantly stronger for PTSD vs. non-traumatized healthy controls as compared to PTSD vs. traumatized controls without PTSD. Finally, episodic memory impairments in PTSD were most pronounced in verbal memory tests as compared to non-verbal memory tests. Our results provide new evidence that PTSD is characterized by impaired episodic long-term memory beyond the traumatic event, and these deficits are particularly pronounced in verbal memory. We will discuss our findings in the context of physiological, psychological and trauma related memory models. From a broader perspective, our findings may have implications for the treatment of PTSD by suggesting that the assessment and, if necessary, training of memory deficits could be included as part of diagnostics and psychotherapeutic treatment.

## Introduction

According to the DSM-5, diagnostic criteria for PTSD include the traumatic event (criteria A), and four other symptom complexes. Importantly, three of these have a direct relationship with memory: intrusion symptoms associated with the traumatic event, such as intrusive memories or flashbacks (criteria B), persistent avoidance of memories and stimuli associated with the traumatic event (criteria C), and negative alterations in cognitions and mood associated with the traumatic event, such as the inability to remember important aspects of the trauma (criteria D). Taking into account the central role of memory, memory related models offer psychological explanations for the development and maintenance of PTSD, and they specifically focus on how traumatic events are being maintained in long-term memory. One central idea is that a traumatic experience long-lastingly changes the structure and function as well as the processing of memory contents (1). However, it remains unclear, whether long-term memory problems in PTSD pertain beyond the episodic event.

Memory impairments may go beyond traumatic events and therefore extend to the general performance of episodic memory as suggested by several studies (2–6). However, others have failed to show such a relationship (7–9). Therefore, Brewin et al. (10) performed a meta-analysis of 27 studies based on 1,472 subjects. Overall, their results indicate impaired verbal and visual memory for emotionally neutral information in PTSD patients compared to controls without PTSD with small to medium effect sizes. Here, stronger effects were found for verbal than visual memory, whereas there were no differences when comparing immediate or delayed retrieval. A year later, Johnsen and Asbjørnsen (11) also published a meta-analysis of 28 studies with 1,489 subjects in which they selectively examined the performance of PTSD patients in verbal memory tests. Their results confirmed Brewin et al. (10) by showing impaired verbal memory performance in PTSD patients compared to controls with a medium effect size. Moreover, they could show that the impairment was greater compared to non-traumatized, healthy controls than compared to trauma-exposed controls without PTSD. Finally, a more recent meta-analysis with 60 studies and 4,108 participants revealed that impairments in PTSD not only include verbal and visual memory but also other cognitive domains especially speed of information processing as well as attention and working memory (12).

A thorough understanding of a possible link between episodic long-term memory and PTSD is important since it may be informative in a clinical context. Indeed, intervention studies could demonstrate a link between episodic memory and the effectiveness of psychotherapeutic treatment of PTSD. For example, patients who did not respond to cognitive behavioral therapy treatment had a significantly poorer verbal memory performance than those who responded to the treatment (13). These differences could not be explained by the initial severity of the disorder, comorbid depression, alcohol or substance abuse, intelligence, performance in attention tests or time since trauma. This suggests that verbal memory deficits reduce the effectiveness of cognitive behavioral therapy, which could also be confirmed for the Eye Movement Desensitization and Reprocessing Therapy (EMDR) (14), Brief Eclectic Psychotherapy (14), and cognitive behavioral therapy for PTSD-associated sleep problems, partly in conjunction with Imagery Rehearsal Therapy (15).

Together, PTSD appears to be characterized by episodic memory problems beyond the traumatic event, which may influence the effectiveness of psychotherapeutic treatment. However, not all studies support such a relationship and recent work (since 2015) on episodic memory impairments in PTSD has not been assessed by metanalytic approaches. Therefore, our overarching research question was whether PTSD patients have impaired episodic memory beyond the traumatic event. To address this issue, we performed a meta-analysis on 47 studies including 3,062 subjects. In a first step, we examined a general relationship between PTSD and episodic memory. That means, no distinction was made between the trauma history of controls or stimulus material in reported memory tests. In a second step, possible effects of these two variables (trauma history and stimulus material) were further tested. Therefore, control subjects were divided into two groups, which were compared separately with the PTSD group: a control group of trauma-exposed people without PTSD (TC) and a group of healthy controls that have never been traumatized (HC). This aimed to address the question whether possible memory deficits in PTSD are related to the disease itself or the experience of a trauma. Finally, in the third step, we examined whether memory impairments in PTSD depend on stimulus material. For this purpose, performances in verbal and non-verbal tests were compared. We hypothesized that (1) PTSD patients show impaired performance in episodic memory tests; (2) PTSD patients show greater deficits in episodic memory compared to non-traumatized, healthy controls (HC) than compared to traumatized controls (TC); (3) compared to controls, PTSD patients show greater impairments in verbal memory than in non-verbal memory.

## Materials and methods

### Literature

The literature review followed the methodological recommendations of the PRISMA statement (Preferred Reporting Items for Systematic Reviews and Meta-Analyses) (16) and was performed by the first author (M.P.). It was primarily based on electronic databases PubMed und Web of Science using the search terms [memory(Title)] OR [cognitive function(Title)] OR [neuropsychological(Title)] AND [posttraumatic stress disorder(Title)] OR [post-traumatic stress disorder(Title)] OR [PTSD(Title)] OR [traumatized(Title)] limited to English and German articles. The database research was complemented by studying the reference lists of existing reviews, meta-analyses and other literature. This study was not pre-registered.

The final search (in May 2020) revealed 782 hits (PubMed: 361, Web of Science: 421). Duplicates were removed, and remaining articles included in a preselection. Another ten potentially suitable studies could be identified through reference lists. On the basis of the title and abstract, apparently irrelevant articles were excluded from the preselection. All remaining studies were checked for suitability based on the following inclusion criteria in full text: They included (a) a group of adults with a current diagnosis of fully developed PTSD based on recognized diagnostic criteria (DSM, or ICD–International Classification of Diseases) and (b) a comparison group of traumatized adult patients without a lifetime PTSD diagnosis (trauma-exposed controls, TC) or without any trauma history (healthy controls, HC), (c) they employed a design that investigated the performance in a test of episodic memory and (d) they provided sufficient statistical parameters of the test results to calculate effect sizes.

### Participants

Comorbid disorders are commonly observed in PTSD and affect 50 to 100% of all PTSD patients (17). Thus, they were not excluded *per se* in the present study, but a purely comorbid group, for example only schizophrenic PTSD patients, is not strictly representative for the population of PTSD patients. Therefore, samples with a psychiatric or neurological comorbidity as a defining criterion were excluded here. The same applies to study samples that specifically examined subjects with traumatic brain injuries (TBI), which is also not uncommon in PTSD. Because of their potential impact on episodic memory (18), the inclusion or exclusion of subjects with TBI was carefully examined. In our present work, publications were only excluded if TBI was a defining criterion of the original study groups (note that inclusion or exclusion of relevant subjects is reported for each study, see below).

If a study included several study groups, and only some met the relevant criteria, these were selectively included in the analysis; those groups that were not suitable were excluded, providing that a comparison of at least one PTSD group and a suitable control group was possible. Studies by the same authors were checked for identical study samples. In such cases, only one of the studies was included.

### Episodic memory tests

We only included studies with tests on episodic long-term memory. In this regard, Pause et al. (19) suggested a delay between encoding and retrieval of least 60 min, since this time is necessary for protein synthesis associated with new episodic long-term memories. However, since most tests used a significantly shorter delay, a more liberal delay of 10 min was chosen here. If the time criterion was met, all types of retrieval (free recall, cued recall, or recognition) were taken into account. Note that, retention-scores and saving-scores (i.e., memory performance after delay minus memory performance when retrieved immediately) were not included, since these were only reported in a few studies and due to conceptual differences from long delay scores.

The distinction of verbal vs. non-verbal memory is simply based on the notion that a test either includes verbal information (e.g., words being presented visually or verbally) or not (e.g., pictures visually presented). Although non-verbal information can often be verbalized (e.g., picture of a tree), several influential memory models assume distinct processing pathways. Most prominently, Baddeley’s model of working memory includes a “phonological loop” and “visuospatial sketchpad”, which process corresponding information, and an interaction between working memory and long-term memory (20). A complete list of all employed memory tests can be found in Table 1.

**TABLE 1:** Overview of studies included in the meta-analysis.

Study	*N* (*n* PTSD/ *n* TC/*n* HC)	Sex	Primary trauma type	Diagnostic criteria	Head trauma exclusion	Memory test	Mean effect size *d*
Bremner et al. (21)	41 (26/–/15)	Men	Military	M-PTSD, SCID-III-R	Yes (any or mild)	Verbal: WMS, SRT Non-verbal: WMS, SRT	−1.13**
Bremner et al. (22)	41 (21/–/20)	Mixed	Interpersonal	ETI, SADS-L	Yes (any or mild)	Verbal: WMS, SRT Non-verbal: WMS, SRT	−0.40
Bremner et al. (23)	43 (18/10/15)	Women	Interpersonal	SCID-IV	Yes (any or mild)	Verbal: WMS Non-verbal: WMS	−0.78*
Carlozzi et al. (24)	64 (21/20/23)	Men	Military	CAPS	No	Verbal: AVLT	−0.28
Crowell et al. (25)	160 (80/160/–)	Men	Military	DIS-III-A, MMPI	Yes (any or mild)	Verbal: CVLT Non-verbal: ROCFT	−0.19
Diener et al. (26)	41 (14/14/13)	Mixed	N/A	DSM-IV-TR	Yes (any or mild)	Verbal: CVLT	−1.31**
Elsesser and Sartory (27)	51 (20/–/31)	Mixed	Mixed	DSM-IV	No	Verbal: RBMT	−0.24
Eren-Koçak et al. (28)	38 (16/22/–)	Mixed	Natural disaster	CAPS	Yes (any or mild)	Verbal: AVLT Non-verbal: ROCFT	−0.39
Geuze et al. (29)	24 (12/12/–)	Men	Military	CAPS, DSM-IV	No	Verbal: Word-Pairs Associates Test	−0.76*
Geuze et al. (30)	50 (25/25/–)	Men	Military	CAPS, SCID-IV	Yes (any or mild)	Verbal: AVLT, CVLT, WMS-R Non-verbal: WMS-R	−0.47
Gilbertson et al. (31)	32 (19/13/–)	Men	Military	SCID-III-R	Yes (any or mild)	Mixed: WMS-R Non-verbal: ROCFT	−0.58
Grigorovich et al. (32)	18 (11/7/–)	Mixed	Electrical injury	PCL-C	Yes (any or mild)	Non-verbal: ROCFT	−1.78**
Gurvits et al. (33)	40 (27/13/–)	Men	Military	SCID-III-R	Yes (significant)	Mixed: WMS-R	−0.15
Gurvits et al. (34)	14 (7/7/–)	Men	Military	CAPS	Yes (any or mild)	Mixed: WMS-R	−0.61
Hori et al. (35)	119 (50/–/69)	Women	Mixed	PDS	No	Mixed: RBANS	−0.66**
Jelinek et al. (36)	80 (40/–/40)	Mixed	Mixed	M.I.N.I., SCID-IV	No	Verbal: PWMT Non-verbal: PWMT	−0.38*
Jelinek et al. (9)	53 (20/24/9)	Mixed	Mixed	SKID-IV	Yes (any or mild)	Verbal: RBMT	0.13
Johnsen et al. (37)	42 (21/21/–)	Mixed	State persecution/terror	CAPS, M.I.N.I.	Yes (significant)	Verbal: CVLT	−0.92*
Koenen et al. (38)	44 (16/28)	Mixed	Mixed	CAPS	Yes (any or mild)	Verbal: WMS-R Non-verbal: WMS-R	−0.29
Koso and Hansen (39)	40 (20/20/–)	Men	Military	DSM-IV	No	Verbal: RBMT Non-verbal: RBMT	−1.33***
LaGarde et al. (4)	38 (21/–/17)	Mixed	Mixed	CAPS	Yes (any or mild)	Verbal: AVLT Non-verbal: AFLT	−0.95*
Lindauer et al. (40)	24 (12/12/–)	Mixed	Mixed	SI-PTSD	Yes (any or mild)	Verbal: CVLT Non-verbal: WMS-R	−0.47
Lipinska et al. (41)	45 (16/15/14)	Women	Interpersonal	CAPS, M.I.N.I.	Yes (any or mild)	Verbal: WMS-III-R	−0.03
Mestrovic et al. (42)	323 (205/118/–)	Men	Military	CAPS, SCID-IV	No	Non-verbal: ROCFT	−0.73***
Narita-Ohtaki et al. (43)	108 (42/–/66)	Women	Mixed	PDS	No	Mixed: RBANS	−0.74***
Neylan et al. (8)	47 (24/23/–)	Men	Military	CAPS	Yes (any or mild)	Verbal: CVLT Non-verbal: WMS-III	0.12
Pederson et al. (44)	51 (17/17/17)	Women	Interpersonal	CAPS	Yes (any or mild)	Verbal: WMS-III Non-verbal: WMS-III	0.01
Pineau et al. (45)	50 (25/–/25)	Mixed	Mixed	SCID-IV	Yes (any or mild)	Verbal: CVLT	−0.48*
Samuelson et al. (46)	68 (37/31/–)	Mixed	Military	CAPS	Yes (any or mild)	Verbal: CVLT Non-verbal: WMS-III	−0.27
Sarac-Hadzihalilović et al. (47)	79 (45/34/–)	N/A	Military	M-PTSD	Yes (any or mild)	Verbal: RBMT Non-verbal: RBMT	−0.83**
Shandera-Ochsner et al. (48)	40 (19/21/–)	Mixed	Military	CAPS	Yes (any or mild)	Verbal: CVLT-II Non-verbal: BVMT-R	−0.50
Shin et al. (49)	53 (30/23/–)	Mixed	State persecution/terror	CAPS	No	Verbal: AVLT Non-verbal: ROCFT	−0.55*
Šodić et al. (50)	48 (25/–/23)	Men	Military	CAPS, ICD-10, MMPI 2, M-PTSD	No	Non-verbal: ROCFT	−1.92***
Stein et al. (51)	61 (17/22/22)	Women	Interpersonal	CAPS, SCID-P	Yes (any or mild)	Verbal: CVLT, VPA, WMS Non-verbal: ROCFT	−0.10
Stricker et al. (52)	171 (92/79/–)	Mixed	Military	CAPS	No	Verbal: CVLT-II Non-verbal: BVMT-R	−0.27*
Sullivan et al. (53)	47 (11/36/–)	Mixed	Military	CAPS	Yes (significant)	Verbal: CVLT, WMS-R Non-verbal: WMS, ROCFT	0.02
Szabó et al. (54)	40 (20/20/–)	Mixed	Mixed	CAPS	No	Mixed: WMS-R	−1.02*
van Liempt et al. (55)	43 (13/15/15)	Men	Military	CAPS	No	Verbal: 15 Word Test	−0.86*
Vasterling et al. (2)	43 (19/24/–)	Mixed	Military	SCID-III-R	Yes (significant)	Verbal: AVLT Non-verbal: CVMT	−0.68*
Vasterling et al. (56)	68 (26/25/17)	Men	Military	SCID-IV	Yes (significant)	Non-verbal: CVMT	0.15
Vasterling et al. (7)	47 (26/21/–)	Men	Military	SCID-IV	Yes (significant)	Non-verbal: CVMT	−0.04
Vythilingam et al. (57)	65 (14/23/28)	Mixed	Military	CES, SCID-IV	Yes (significant)	Verbal: WMS-R, SRT Non-verbal: WMS-R, SRT	−0.48
Woodward et al. (58)	95 (48/47/–)	N/A	Military	CAPS	No	Verbal: WMS-III	−0.56*
Wrocklage et al. (59)	84 (44/40/–)	Mixed	Military	CAPS	Yes (significant)	Verbal: CVLT-II Non-verbal: ROCFT	−0.06
Yehuda et al. (60)	102 (36/26/40)	Mixed	Mixed	CAPS, SCID-IV	Yes (significant)	Verbal: CVLT	−0.56*
Yehuda et al. (3)	65 (30/20/15)	Men	Military	CAPS, SCID-IV	Yes (significant)	Verbal: CVLT	−0.79*
Zlomuzica et al. (6)	42 (21/21)	Mixed	N/A	Mini-DIPS	Yes (any or mild)	Verbal: RBMT Non-verbal: RBMT	−0.06

AFLT, Aggie Figures Learning Test; AVLT, Auditory Verbal Learning Test; CAPS, Clinician Administered PTSD Scale; CES, Combat Exposure Scale; CVLT, California Verbal Learning Test; DIS-III-A, Diagnostic Interview Schedule, Version III-A; DSM-IV, Diagnostic and Statistical Manual of Mental Disorders, Fourth Edition; DSM-IV-TR, Diagnostic and Statistical Manual of Mental Disorders, Fourth Edition, Text Revision; ETI, Early Trauma Inventory; ICD-10, International Classification of Diseases, 10th Revision; M.I.N.I., Mini International Neuropsychiatric Interview; Mini-DIPS, Diagnostic Interview for Mental Disorders–Short Version, German Version; MMPI, Minnesota Multiphasic Personality Inventory; M-PTSD, Mississippi Scale for Combat related PTSD; PCL-C, Posttraumatic Symptom Checklist, Civilian Version; PDS, Posttraumatic Diagnostic Scale; PWMT, Picture Word Memory Test; RBANS, Repeatable Battery for the Assessment of Neuropsychological Status; RBMT, Rivermead Behavioral Memory Test; ROCFT, Rey-Osterreith Complex Figure Test; SADS-L, Schedule for Affective Disorders and Schizophrenia–Lifetime Version; SCID-III-R, Structured Clinical Interview for DSM-III-R; SCID-IV, Structured Clinical Interview for DSM-IV; SKID-IV, Structured Clinical Interview for DSM-IV, German Version; SCID-P, PTSD module of the Structured Clinical Interview for DSM-IV; SI-PTSD, Structured Interview for Posttraumatic Stress Disorder; SRT, Selective Reminding Test; WMS, Wechsler Memory Scale; WMS-III, Wechsler Memory Scale–Third Edition; WMS-III-R, Wechsler Memory Scale–Third Edition Revised; WMS-R, Wechsler Memory Scale–Revised.

*p ≤ 0.05, **p < 0.001, ***p < 0.0001.

Studies that used emotionally valent, in particular trauma-associated stimuli within the test session, or otherwise provoked the elicitation of symptoms or memories of the trauma (61) were excluded. The same is true for studies with interventions (e.g., drug-based) that could potentially impact on memory performance.

### Meta-analyses

#### Methodological approach

To answer our research questions, several meta-analyses were performed. The first hypothesis of a general negative relationship between PTSD and episodic memory was tested on all studies by comparing the group of PTSD patients with a group of all control subjects without PTSD across all episodic memory tests.

To test our other hypotheses, separate meta-analyses were carried out on a part of the data using different control groups (all controls vs. TC vs. HC) and stimulus material (all tests vs. verbal tests vs. non-verbal tests). All studies that allowed a corresponding comparison were included in the respective analyses. For every possible combination, an overall effect was determined across all studies and then compared using a comparison of means. To test our second hypothesis (greater episodic memory impairments in PTSD vs. HC than PTSD vs. TC), a statistical comparison of the overall effects for PTSD vs. TC with PTSD vs. HC was carried out independently of stimulus material. To test the third hypothesis (greater impairments in PTSD in verbal vs. non-verbal memory), the overall effects of all verbal tests were compared with those of all non-verbal tests.

#### Effect size calculation–Cohen’s *d*

In a first step, an effect size (Cohen’s *d*) was determined for each original study, which was subsequently used in the respective meta-analysis in order to calculate the mean overall effect across all studies. Specifically, for each episodic memory test in each study, a Cohen’s *d* (bias corrected for small samples) was calculated (62). In three studies no mean values and standard deviations were reported; therefore, Cohen’s *d* was calculated on the basis of *t*-, and *F*-values (63) and *X*^2^ values (64).

Several studies provided more than one effect size per group, e.g., on the basis of different test results or test indices such as free recall and cued recall. To avoid an imbalanced impact of these studies, corresponding effect sizes were averaged; as a result, each study was included with one effect size per meta-analysis.

In studies that report test data for both trauma-exposed and non-traumatized control groups, combined sample sizes, mean values and standard deviations were calculated (65), which were then used in our meta-analyses that did not differentiate between both groups. This approach avoids that the PTSD group is being entered into the analysis twice with the same values. The effect size was then derived from the mean difference between the combined measures of the control groups and the simple measures of the PTSD group according to the methodology described above.

A negative Cohen’s d indicates a poorer test performance in the PTSD group compared to the control group, and a positive Cohen’s d indicates a better test performance in the PTSD group compared to the control group. Values of 0.2, 0.5, and 0.8 can be interpreted as small, medium and large effect, respectively (66).

Effect sizes may depend on several variables including the type of memory test (i.e., free recall, cued recall, and recognition), retrieval delay, trauma type, age, gender, education or ethnicity and should therefore be included in the analyses. However, further disentangling such relationships is not possible here since the necessary information was not provided by all original studies.

#### Random-effects meta-analysis

Random-effects meta-analyses were carried out according to Borenstein et al. (65). Here, the observed effect sizes from individual studies are weighted by the inverse of the total variance of that study, which ensures that the overall effect is as precise as possible. The final significance testing was based on Z-scores (derived from weighted overall effect sizes) and one-sided testing (given our *a priori* hypotheses, see introduction). Apart from effect sizes (d*) and Z-scores, we also report one-sided confidence intervals (CI) and *I*^2^. The latter is a measure of heterogeneity (67, 68) and quantifies the degree of inconsistency across studies by providing direct information about the percentage of heterogeneity of the total variance of the primary studies. It ranges from 0 (homogeneity) to 100% (strong heterogeneity) and can be compared directly between different meta-analyses regardless of the number of studies included or the outcome variable (67). Values of 25, 50 and 75% indicate low, moderate and high heterogeneity (67).

## Results

### Overview of systematic literature research

**FIGURE 1:**
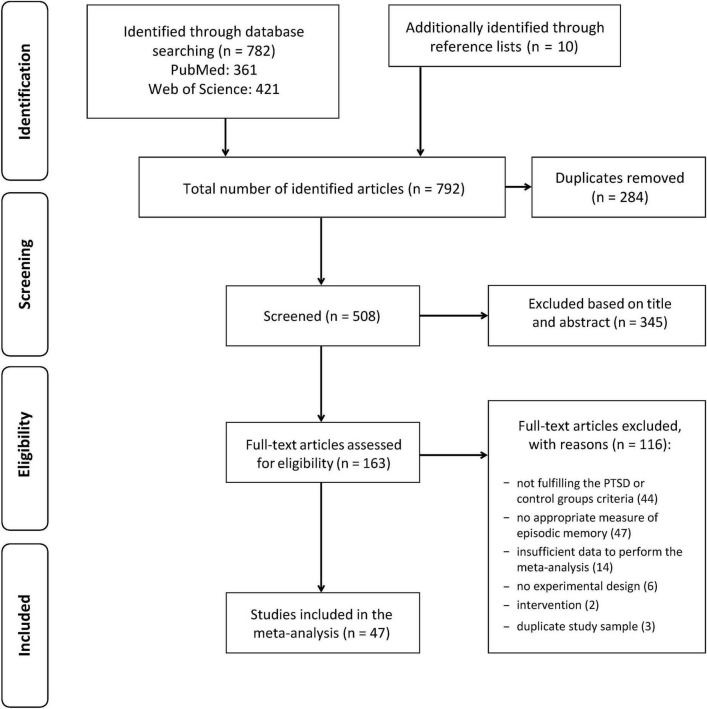
Flow chart of literature search and selection process.

Figure 1 summarizes the process of literature research and literature selection using the PRISMA flow chart. In the end, 47 studies were suitable and therefore included in the meta-analysis. Table 1 gives an overview of the included studies, sample characteristics, measures used to diagnose PTSD, type of trauma and status of inclusion or exclusion of subjects with head trauma. It also provides an overview of the employed memory tests, differentiated into verbal and non-verbal tests, and it shows the mean effect-size (Cohen’s *d*) per study on the basis of a comparison between PTSD vs. control subjects averaged across all memory tests used.

### Studies and sample characteristics

The 47 studies (Table 1) include a total of 3,062 subjects, including 1,419 PTSD patients, 1,060 trauma-exposed controls, 534 healthy controls without trauma history, and 49 control subjects who could not clearly be assigned to one of the two control groups (TC or HC); therefore, they were only included in calculations that did not distinguish between both control groups.

Sixteen of the studies included only men, six included only women, two did not report the sex of their participants, and the majority of the studies (23) included male and female samples. All participants in our total sample are at least 18 years old, and the mean weighted age (based on sample size) was 38.75 years. Three studies (46, 47, 53) did not provide any information on the exact age; therefore, these studies were not included in the calculation of the mean age. Only three studies examined older subjects with an average of over 65 years. Specifically, Jelinek et al. (9) included displaced children in World War II (*M* = 71.15 years), Yehuda et al. (60) examined Holocaust survivors (*M* = 68.91 years) and Yehuda et al. (3) investigated older veterans (*M* = 66.06 years).

The majority of studies (25 studies) included veterans who have experienced military trauma. Eleven studies included samples of various types of primary trauma. The remaining studies examined individuals who have experienced interpersonal trauma (five studies), state persecution or terror (two studies), natural disasters (one study), or who have suffered from electrical injuries (one study). Two studies do not provide any information on the type of trauma. In most studies, PTSD was diagnosed using the Clinician Administered PTSD Scale (CAPS, 25 studies) and/or the Structured Clinical Interview for DSM (SCID, 16 studies). The other diagnostic instruments used are shown in Table 1.

Twenty-three studies excluded subjects with any or mild head trauma (or loss of consciousness for 15 min), ten with severe head trauma (or loss of consciousness for 30 min), and 14 studies did not report the exclusion of these kind of subjects.

To investigate episodic memory performance, 36 studies used verbal and 30 studies used non-verbal memory tests. In six studies, only one overall measure was provided for some tests, which includes both verbal and non-verbal subtests; therefore, these indices were only included in analyses that did not distinguish between stimulus material. The most frequently used memory tests are the Wechsler Memory Scale (18 studies; verbal and non-verbal subtests), California Verbal Learning Test (CVLT, 15 studies; verbal) and Rey-Osterrieth complex figure test (ROCFT, 10 studies; non-verbal), sometimes in different versions. The mean effect sizes in individual studies (including all tests and control groups) varied between *d* = −1.919 and *d* = 0.154. According to Cohen (66), this corresponds to very strong negative to weak positive effects.

### Relationship between post-traumatic stress disorder and episodic memory

Table 2 shows an overview of the results of the meta-analyses for the comparison PTSD vs. all controls, and for the separate comparisons PTSD vs. TC and PTSD vs. HC. The “overall analysis” for the three groups includes all memory tests (i.e., including verbal and non-verbal tests). The results of the sub-analyses (i.e., one for verbal and one for non-verbal tests) are listed below. All tests reached statistical significance and indicated a poorer performance of the PTSD group compared to the control group. The proportion of variance in heterogeneity (*I*^2^) varies between 38 and 77% depending on the analysis, indicating low to high heterogeneity.

**TABLE 2:** Results of random-effects meta-analyses by control group and stimulus material.

Analysis	Number of studies *(k)*	Number of subjects *(n)*	Mean weighted effect size (d*)	95% confidence interval	Z-score (significance of effect size)	*I^2^ (%)*
*PTSD vs. all controls*						
overall analysis	47	3,062	−0.50	−∞ to −0.40	−8.405***	54
verbal	36	2,205	−0.47	−∞ to −0.36	−7.075***	50
non-verbal	30	2,039	−0.40	−∞ to −0.26	−4.771***	65
*PTSD vs. TC*						
overall analysis	36	2,172	−0.42	−∞ to −0.31	−6.382***	46
verbal	28	1,607	−0.38	−∞ to −0.27	−5.520***	38
non-verbal	24	1,677	−0.35	−∞ to −0.21	−3.965***	61
*PTSD vs. HC*						
overall analysis	21	1,046	−0.60	−∞ to −0.42	−5.640***	60
verbal	17	728	−0.65	−∞ to −0.45	−5.312***	57
non-verbal	10	388	−0.42	−∞ to −0.05	−1.872*	77

*p < 0.05, ***p < 0.0001.

### General impairment in episodic memory in post-traumatic stress disorder

**FIGURE 2:**
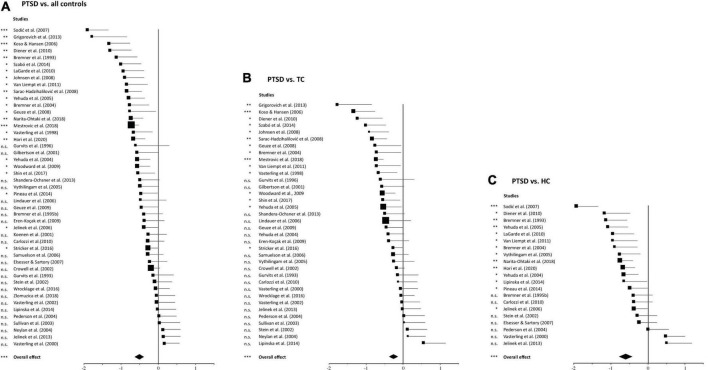
Forest plots of group comparisons. **(A)** Comparison of post-traumatic stress disorder patients with all controls, **(B)** comparison of PTSD patients with traumatized controls, **(C)** comparison of PTSD patients with non-traumatized healthy controls. Squares in the forest plot represent the individual effect sizes of the primary studies, and square size corresponds to the relative size of the sample studied. For each comparison, the diamond at the bottom indicates the overall effect of the meta-analysis. The width of the diamond and the horizontal lines extending from the squares represent the respective confidence intervals. n.s. abbreviates not significant. *p ≤ 0.05, ^**^p < 0.001, ^***^p < 0.0001.

The overall analysis, including all memory tests and control groups from all 47 studies, shows a highly significant lower episodic memory performance in the PTSD group compared to controls (d* = −0.50, *p* < 0.0001), which confirms our first hypothesis. The size of d* is in the medium range, with the associated confidence interval reaching into the range of a weak effect. The heterogeneity analysis indicates moderate heterogeneity between the included studies (*I*^2^ = 54%). Figure 2A shows the effect sizes for the overall comparison using a forest plot. The squares symbolize the effect sizes of each individual study and their one-sided 95% confidence interval, represented by the horizontal lines to the right. The diamond represents the mean, weighted overall effect.

In almost all studies (Figure 2A and Table 1), the effects are in the expected negative direction and two studies (32, 50) showed a particularly pronounced negative effect (at the top of Figure 2A). In order to rule out that the overall effect was specifically driven by these two studies, the analysis was run again without them. It also revealed a statistically significant overall effect [d* = −0.46, *p* < 0.0001, 95% CI (−∞, −0.360)] again indicating that episodic memory was, across all studies, reduced in the PTSD groups. In contrast, five studies revealed a weak positive effect suggesting that the PTSD groups performed better than the controls. However, none of these five effects were statistically significant. Out of the 42 studies with a negative effect, 23 were statistically significant. Finally, across all studies there is a relatively large distribution of effect sizes and a tendency toward broad confidence intervals, which confirms the prior assumption of existing heterogeneity of our random-effects model.

### Stronger memory impairments in post-traumatic stress disorder vs. healthy controls

**FIGURE 3:**
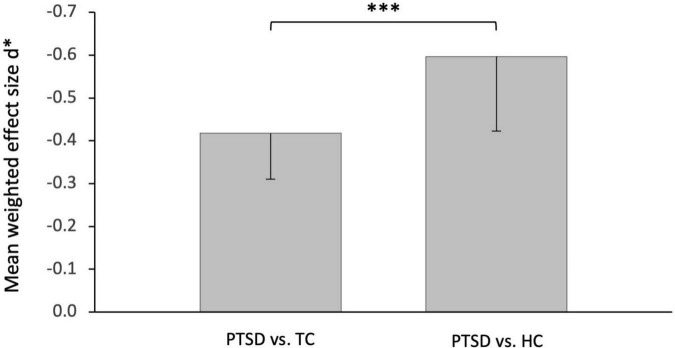
Effect of the control group. Significantly greater impairment of episodic memory in post-traumatic stress disorder patients compared with non-traumatized healthy controls (HC, **right**) than compared with trauma-exposed controls (TC, **left**). ^***^p < 0.0001.

Separate meta-analyses of the two control groups found greater effects for the comparison PTSD vs. HC (d* = −0.60, *p* < 0.0001, Figure 2C) than for the comparison PTSD vs. TC (d* = −0.42, *p* < 0.0001; Figure 2B). The statistical comparison of these two overall effects (Figure 3) showed a very strong, highly significant effect [*d* = −2.15, *p* < 0.0001, 95% CI (−∞, −1.59)]. This confirms our second hypothesis of a significantly stronger memory impairment in PTSD in comparison to healthy controls than compared to trauma-exposed controls.

### Stronger memory impairments in verbal memory in post-traumatic stress disorder

**FIGURE 4:**
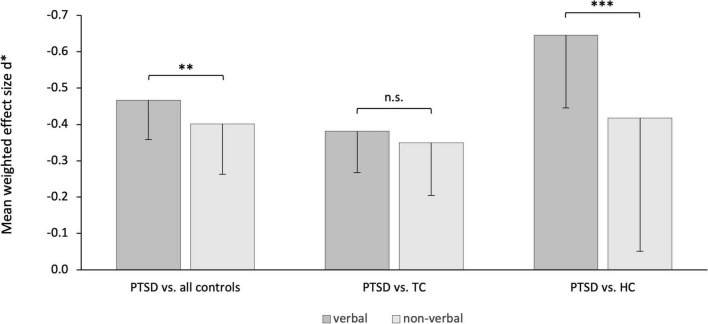
Effect of stimulus material separated by analysis groups. Stronger impairments of episodic memory of post-traumatic stress disorder patients in verbal than non-verbal memory tests. The effect was statistically significant for the comparison with all controls **(left)** and in sub-comparison with healthy controls (HC, **right**) but not compared to trauma control group (TC, **middle**). n.s. abbreviates not significant. ^**^p < 0.001, ^***^p < 0.0001.

Separate analyses of the performance in verbal vs. non-verbal memory tests in PTSD patients vs. controls revealed a statistically significant effect [*d* = −0.86, *p* < 0.001, 95% CI (−∞, −0.439)]. Specifically, PTSD patients showed more severe impairments in verbal memory tests (d* = −0.47, *p* < 0.0001) than in non-verbal memory tests (d* = −0.40, *p* < 0.0001; Figure 4 left panel). In two separate analyses, the effect for the comparison PTSD vs. HC remained significant [*d* = −1.34, *p* < 0.0001, 95% CI (−∞, −0.83), Figure 4, right panel], but not for the comparison PTSD vs. TC [*d* = −0.40, *p* = 0.08, 95% CI (−∞, 0.067), Figure 4, middle panel].

## Discussion

We investigated the relationship between PTSD and episodic long-term memory performance. Therefore, several random effects meta-analyses were carried out on the basis of 47 studies and 3,062 adult human subjects, including PTSD patients, traumatized people without PTSD and non-traumatized healthy controls. Our findings demonstrate (a) impaired episodic long-term memory in PTSD patients compared to all controls, (b) this effect is more pronounced compared to non-traumatized healthy controls than compared to traumatized controls, and (c) the impairment is more pronounced in verbal than in non-verbal memory tests. As such, our findings provide further evidence that episodic long-term memory problems beyond the traumatic event are a hallmark of PTSD. Although this includes verbal and non-verbal information, it is more pronounced for verbally accessible memories further indicating a domain specific dissociation. Moreover, the experience of a trauma alone seems to lead to deficits in memory performance, but the disease of PTSD is associated with even greater impairments.

Our first observation of episodic long-term memory deficits in PTSD beyond the traumatic event confirms previous meta-analyses (10–12), now including several more recent studies. While meta-analyses can only speculate about the underlying mechanisms, other work gives more specific hints, including neuroanatomical changes of the hippocampus, altered sleep, inefficient learning strategies and interferences through PTSD symptoms. All four explanations are not mutually exclusive and will further be discussed below.

Physiological models assume that PTSD leads to structural brain changes, especially the hippocampus, that is particularly important for long-term memory. This could be caused by high concentrations of glucocorticoids, such as cortisol, which are released during stressful events, leading to damage of hippocampal neurons (69). Evidence is provided by empirical work demonstrating reduced hippocampus volumes in PTSD patients compared to controls (22, 34, 70), which could even be confirmed in a meta-analysis with a total of 1,868 subjects across several studies (71). However, since most studies are cross-sectional, smaller hippocampi could also be a risk factor of PTSD (70) instead of a consequence. While the direction of the effect remains to be determined (see below), for instance in longitudinal studies, a role of the hippocampus in PTSD related memory problems appears reasonable.

Differences in hippocampus volume could also help to explain our finding of more pronounced verbal compared to non-verbal memory deficits in PTSD. Specifically, some studies found a significant volume reduction in the left but not right hippocampus (72, 73). While the left hippocampus is particularly relevant for verbal memory, the right hippocampus is more related to visuospatial memory performance (74, 75). However, these findings are inconclusive since the opposite pattern, namely significant volume reductions only in the right but not left hippocampus in PTSD, has also been reported (22). In military veterans with PTSD, both hippocampi were significantly reduced, but when taking into account the extent of combat operations, only the left hippocampus remained significantly smaller (34). Although this relationship was unaffected by alcohol abuse (34), others suggest that alcohol related disorders are a common comorbidity of PTSD (76) with potentially aversive effects on the hippocampus (77). Together, these findings indicate that anatomical brain changes, especially in the hippocampus, are related to episodic memory deficits in PTSD. However, future studies should further investigate whether structures responsible for processing verbal memory are more severely affected.

Impaired episodic memory in PTDS might also relate to changes in sleep. In fact, sleep disorders and reduced sleep quality are part of the clinical picture of PTSD (78), which might have a negative impact on consolidation of novel information and therefore subsequent memory retrieval. For instance, PTSD patients showed a more fragmentated REM sleep (rapid eye movement), which was the only significant predictor of impaired memory retention after sleep; such a relationship was not found in healthy controls or a trauma control group without PTSD (41). While these studies clearly suggest that changes in sleep-related consolidation closely relate to episodic memory impairments in PTSD, they cannot explain the full picture. Specifically, only two studies in the present meta-analysis included a sleep phase between learning and retrieval (41, 55) while all others did not. Since memory consolidation may not necessarily require sleep (79, 80), future studies may investigate the impact of changes in sleep and sleep independent rapid systems consolidation in PTSD.

Ineffective learning strategies in PTSD patients could also explain memory deficits. For instance, a specific relationship between changes in learning strategies and impairments of executive functioning in PTSD was reported, which could further be linked to deficits in verbal memory (81). Accordingly, PTSD patients used serial organization to a lesser extent (i.e., retrieval of information in the order as it was presented during learning), but showed more pronounced recency effects (i.e., enhanced memory for items presented last in a list during encoding) and made more intrusion errors (i.e., incorrect memory for items not belonging to a word list) than controls. However, more work is needed here since the relationship between memory impairments and learning strategies might be confounded by symptoms of depression (81).

Along these lines, PTSD symptoms, such as intrusions, avoidance and hyperarousal, might interfere with processes involved in episodic memory (82). Accordingly, these symptoms excessively bind cognitive resources, which are therefore no longer available for encoding, consolidation or retrieval of memories. Indeed, empirical evidence shows that the extent of intrusions in PTSD was associated with performance in a verbal memory test (11). There is also evidence of a negative relationship between the size of the hippocampus and some PTSD symptoms such as reexperiencing symptoms (83) and dissociation (73).

Despite the apparent links between episodic memory impairments in PTSD and several factors mentioned above, the causal relationship is less clear. On the one hand, PTSD could lead to deficits in episodic memory. On the other hand, it could be possible that deficits in episodic memory, that already existed before the trauma, increase the likelihood of developing PTSD. Already existing memory problems before PTSD would, therefore, be a predictor or factor of vulnerability of the disorder. Finally, other factors, for example trauma-related, sociodemographic or personality-related factors, which promote the development of PTSD when experiencing a trauma, could lead to memory impairments and thus may act as moderators. The literature provides evidence in favor of all three accounts.

The first account of a direct consequence of memory deficits through PTSD is supported by a study investigating recently traumatized people with high vs. low PTSD symptoms (84). There were no significant differences between both groups in “long delay” scores of verbal and non-verbal memory tests indicating no link between early PTSD symptoms and episodic memory performance. The findings, therefore, indicate–with all due cautions–that memory deficits develop throughout the course of PTSD.

The second account, suggesting that deficits in episodic memory are a predictor of PTSD, is supported by a large-scale epidemiological longitudinal study that examined young adults before and after a devastating natural disaster (85). It revealed an inverse relationship between the development of PTSD symptoms (including intrusion and arousal) and verbal episodic memory performance in the CVLT three years before the trauma. In other words, better pre-traumatic verbal memory was associated with lower probability of developing PTSD symptoms after the trauma, even when controlling for severity of the trauma, depressive symptoms, alcohol consumption and other factors. Further support comes from a study on monozygous twin pairs suggesting that memory impairments constitute a predisposing factor for the development of PTSD (34). Accordingly, the healthy twin, who was not exposed to trauma, showed the same performance in a verbal memory test as the trauma-exposed twin with PTSD. Further, this performance was significantly worse compared to trauma-exposed individuals without PTSD and their non-traumatized twin brothers.

Nixon et al. (86) provide evidence for the third account of a moderator hypothesis. They examined 73 rape victims with full (92%) or subthreshold PTSD (i.e., not all criteria for a PTSD diagnosis were met in 8%), and reported that only the severity of previous traumatic experiences predicted delayed episodic memory but not PTSD symptoms, depression scores, or intelligence (i.e., IQ). In other words, only the severity of the trauma correlated negatively with episodic memory performance. Since higher trauma severity is also a risk factor for the development of PTSD (87), it is possible that PTSD patients who experienced particularly severe traumatic experiences, more likely developed PTSD and episodic memory deficits.

Together, various factors appear to be involved in the development of episodic memory deficits in PTSD and, therefore, several aspects of learning and memory may be affected (i.e., encoding, consolidation, and retrieval). Future studies should employ longitudinal designs, covering both the pre-and post-traumatic period, in order to shed more light on their exact relationship.

Understand the exact nature of episodic memory deficits in PTSD is particularly important since they might limit the effectiveness of psychotherapy (13–15). A key aspect of many trauma therapies is trauma confrontation. Here, trauma related memories are retrieved in a protected therapeutic environment, modified or extended in order to integrate them into their autobiographical context at the time. Since this requires the ability to precisely recall details of the traumatic event, to form new memories and integrate novel information, deficits in episodic memory should be taken into account when treating PTSD with psychotherapy. This is particularly relevant given the pronounced deficits in verbal memory, since psychotherapy typically involves a large amount of verbal conversations. Therefore, assessment and possibly treatment of episodic memory deficits, using e.g., memory trainings, could be an important part of the diagnosis as well as the development and implementation of individual treatments (6, 82, 88). Preliminary evidence that memory training can successfully contribute to a reduction in PTSD symptoms already exists (89) but this needs to be further investigated more directly.

Our findings largely confirm previous meta-analyses (10–12), but they also tap into more specific research questions and they include several new studies. For instance, we specifically focused on long delay scores and excluded retention scores, which differs from Scott et al. (12). Johnsen and Asbjørnsen (11) compared both traumatized vs. non-traumatized but they only included verbal memory, and Scott et al. (12) did not distinguish between trauma groups in their memory analysis. Moreover, in comparison to the most recent meta-analysis, we included seven new studies since 2015 but (due to different inclusion criteria, see “Materials and methods”) we included 17 studies that were not part of the work by Scott et al. (12). Moreover, in the light of the replication crisis (90, 91), it is important to re-analyze data with different approaches. Therefore, by replicating previous findings with other methods and novel data, our work provides novel insights and, at the same time, further empirical evidence for impaired memory abilities in PTSD beyond the traumatic event.

Meta-analyses have several advantages but also limitations. On the one hand, they allow us to include various studies and, in this case, a large number of patients, which increases statistical power. On the other hand, there is a tendency in research not to publish non-significant results (publication bias), which can lead to an incomplete view of the actual state of research and thus falsify the results of a meta-analysis. Such an influence on the results cannot be excluded in the present work either. In addition, the included studies are heterogenous with respect to several potentially important aspects. Specifically, patients differed in their primary trauma type (Table 1), which may be associated with differences in severity of the trauma, treatment history, or responsiveness to clinical treatments. Furthermore, and as mentioned above, effect sizes may depend on other factors such as the type of memory test, retrieval delay, age, gender, education, or ethnicity. These should be more thoroughly provided in future work to allow a systematic investigation of their impact on long-term memory impairments in PTSD.

To summarize, the present meta-analysis on the relationship between PTSD and episodic memory includes 47 studies and 3,062 subjects. Our results demonstrate that the experience of a trauma alone is already associated with episodic memory deficits beyond the trauma event, and PTSD is further associated with even greater impairments. Although verbal and non-verbal memory is affected, greater deficits for verbal memory content argue in favor of domain specific effects. Further, our findings have implications for psychotherapeutic treatments of PTSD by suggesting that assessing and, if necessary training of memory deficits, should be included in the diagnosis and treatment plan.

## Data availability statement

All data and analysis code are available from the corresponding author upon request, including a formal project outline.

## Author contributions

MP analyzed the data. Both authors designed the study, wrote the article, and approved the final version of the manuscript for submission.
